# Crosstalk between the p38 and TGF-β signaling pathways through TβRI, TβRII and Smad3 expression in plancental choriocarcinoma JEG-3 cells

**DOI:** 10.3892/ol.2014.2255

**Published:** 2014-06-16

**Authors:** YUSI TAN, QIAN XU, YUHONG LI, XIAODAN MAO, KONGYAN ZHANG

**Affiliations:** Department of Basic Medicine, Chengde Medical College, Chengde, Hebei 067000, P.R. China

**Keywords:** p38 MAPK, TGF-β, TβRI, TβRII, Smad3, choriocarcinoma

## Abstract

Choriocarcinoma is a highly aggressive tumor that develops from germ cells. Some choriocarcinomas originate in the testes or ovaries, while others may develop in the uterus after a normal pregnancy or after miscarriage. The tumor is characterized by early hematogenous spread to distal organs, such as the lung and brain. Transforming growth factor β1 (TGF-β1) is key in regulating tumor cell proliferation and invasion through a variety of Smad-dependent and -independent pathways, including the p38 mitogen-activated protein kinase (MAPK) pathway. There appears to be crosstalk between the TGF-β/Smad and p38 MAPK pathways; however, the molecular mechanisms underlying the crosstalk are not fully understood. The present study validated the role of TGF-β signaling in cancer progression and explored the interaction between Smad and p38 MAPK signaling on transduction mediators in choriocarcinoma using the JEG-3 cell line. MTT assay was used to detect the effect of TGF-β1 on JEG-3 cell proliferation. Cells were treated with p38 MAPK inhibitor and TGF-β receptor inhibitor, followed by TGF-β1, and reverse transcription quantitative real-time polymerase chain reaction was used to examine the transcriptional levels of Smad3 and TGF-β receptors. The data demonstrated that TGF-β can enhance the viability of JEG-3 cells. Blockade of the TGF-β and p38 MAPK pathways attenuated the expression of Smad3, TGF-β receptor type I (TβRI) and TβRII, and inhibited their expression in a dose-dependent manner. Analysis revealed that p38 MAPK is involved in and contributes to the TGF-β pathway, dependent on the regulation of TβRI, TβRII and Smad3. Further investigation of the interactions between the TGF-β and p38 MAPK pathways may offer potential venues for therapeutic intervention for choriocarcinoma.

## Introduction

Choriocarcinoma, which is a gestational trophoblastic disease, is a highly malignant trophoblastic tumor. It arises almost exclusively in the placenta of pregnant women and may occur even after a normal pregnancy (1). The embryonic trophocyte loses its original structure, and the abnormal tissue invades the muscular layer of uterus and then further spreads to other organs through the venous and lymphatic systems (2).

Transforming growth factor β (TGF-β), which belongs to a growth factor super-family, is a potent regulator of tumor growth (3). TGF-β is a multifunctional polypeptide cytokine and it regulates a variety of cellular processes, such as cell cycle arrest, differentiation, proliferation, extracellular matrix (ECM) production, the promotion of ECM formation and the suppression of immune response (4,5). Previous studies have demonstrated that TGF-β1 has an inhibitory effect at the first stage of tumorigenesis, but certain late-stage tumor cells escape this cytostatic effect (6–8). Compared with other tumor types, the role of the TGF-β/Smad signaling pathway in the development and proliferation of placental choriocarcinoma has rarely been investigated.

TGF-β exerts its biological function through the TGF-β/Smad pathway, which initiates by binding to its serine and threonine kinase receptors, the TGF-β receptor type II (TβRII) and TβRI, on the cell membrane (9). TβRII firstly phosphorylates and activates TβRI, then forms a complex with TβRI. The receptor complex recruits and phosphorylates the R-Smad proteins, Smad2/3, via phosphorylation at their C-terminal serine residues (10). Thus, the signal crosses the membrane to the inside of the cell. Smad4 (Co-Smad) works as a mediator by transporting phosphorylated R-Smad (Smad2 and Smad3) into the nucleus, where target genes are processed (11,12). Any mutations of components in the TGF-β signaling pathway contribute to the loss of TGF-β1 growth control in cancer (13).

In addition to this Smad2/3 pathway, TGF-β has been reported to activate other signaling molecules, such as mitogen-activated protein kinases (MAPKs) (14). MAPKs consist of four distinct groups: The extracellular signal-related kinases (ERKs), the c-jun N-terminal kinases, the atypical MAPKs (ERK3, ERK5, and ERK8) and the p38 MAPKs (15,16). The p38 pathway, like other MAPK pathways, features a cascade of protein kinases, culminating in the phosphorylation of p38 MAPK on specific threonine and tyrosine residues (17). The phosphorylation is mediated primarily by upstream MKK3 and MKK6. MKK3 and MKK6 in turn are regulated by phosphorylation through upstream MAPK kinase kinases (18,19). The p38 MAPK pathway is responsive to environmental stresses (UV, ionizing radiation, oxidative stress and FAS ligands) and inflammatory cytokines (20). Our previous study demonstrated that blocking the TGF-β pathway by using a TGF-β receptor inhibitor significantly reduces the expression levels of p38 and phospho-p38 in JEG-3 cells. Additionally, treatment of JEG-3 cells with a p38 MAPK inhibitor (SB 203580) attenuated TGF-β1-induced Smad3 protein expression and suppressed the activation of Smad3 (21). These results suggested that there is crosstalk between p38 MAPK and Smad3 through TGF-β signaling in human choriocarcinoma.

TGF-β receptors are the gateways of the intracellular signaling. The receptor complex is a central point for protein interactions; post-translational modifications may have key functions in the transduction of TGF-β signals (22). A previous study suggested that inhibition of TβRI activity blocked TGF-β-induced MAPK activation (23). According to a study by Bandyopadhyay *et al*, differences in the level of TβRII expression determined whether or not TGF-β activated or inhibited ERK1/2, and TβRII alone was able to mediate TGF-β signaling to ERK1/2 without participation of TβRI/Alk5 (5). TGF-β-mediated MAPK activation has been reported to be associated with tyrosine phosphorylation of TGF-β receptors (24). Therefore, the present study aimed to investigate whether there is an association between TGF-β receptors and p38 MAPK in choriocarcinoma. The study focused on the interaction between the p38 MAPK signaling pathway and TGF-β receptors through TGF-β1 stimulation in human choriocarcinoma cells.

## Materials and methods

### Materials

The human placental choriocarcinoma JEG-3 cell line was obtained from the State Key Laboratory of Reproductive Biology, Institute of Zoology, Chinese Academy of Sciences (Beijing, China). The study was approved by the ethics committee of the Natural Science Foundation of Hebei Province and the Education Department of Hebei Province, Hebei, China.

### JEG-3 cell culture

JEG-3 cells were grown in an incubator, with 5% CO_2_ at 37°C, in RPMI-1640 supplemented with 10% fetal bovine serum (FBS; Hangzhou Sijiqing Biological Engineering Materials Co., Ltd, Hangzhou, China), 100 mM sodium pyruvate, 200 mM glutamine, 100 μg/ml streptomycin and 100 U/ml penicillin. When the cells reached ~80% confluency, they were subcultured with 0.25% trypsin and 0.02% EDTA.

### MTT assay

After reaching 80% confluency, cells were trypsinized and cultured in 96-well plates with an initial concentration of 3×10^4^/ml in RPMI-1640 containing 10% FBS per well. After 24 h culturing, the medium was changed to RPMI-1640 without FBS, and cells were cultured for a further 12 h to ensure cell synchronization. A total of 42 wells were divided into seven groups as follows: Blank, control, and 1-, 2-, 6-, 12- and 24-h groups. TGF-β1 (PeproTech, Inc., Rocky Hill, NJ, USA), at a concentration of 5 ng/ml, was added to all wells of the plate, with the exception of those for the blank and control groups. Cells in the control group were cultured with RPMI-1640, while the wells for the blank group were filled with phosphate-buffed saline only. Each specimen was prepared in four replicates. After treatment with TGF-β1 for 1, 2, 6, 12 and 24 h, the medium was removed from wells in the 1-, 2-, 6-, 12- and 24-h groups, respectively. Next, 180 μl RPMI-1640 supplemented with 20 μl of 5 ng/ml MTT (Sigma-Aldrich, St. Louis, MO, USA) in phosphate buffered saline was added to each of these wells, and the plate was incubated at 37°C for 4 h. Following the incubation, the medium was removed and 150 μl of dimethyl sulfoxide (Sigma-Aldrich) per dish was added. Following agitation for 10 min at room temperature, the absorbance of each group was assayed at 490 nm with an enzyme-linked immunosorbent assay plate reader (Multiskan MK3; Thermo Labsystems Oy, Helsinki, Finland).

### Reverse transcription quantitative real-time polymerase chain reaction (RT-qPCR)

**Figure 1:**
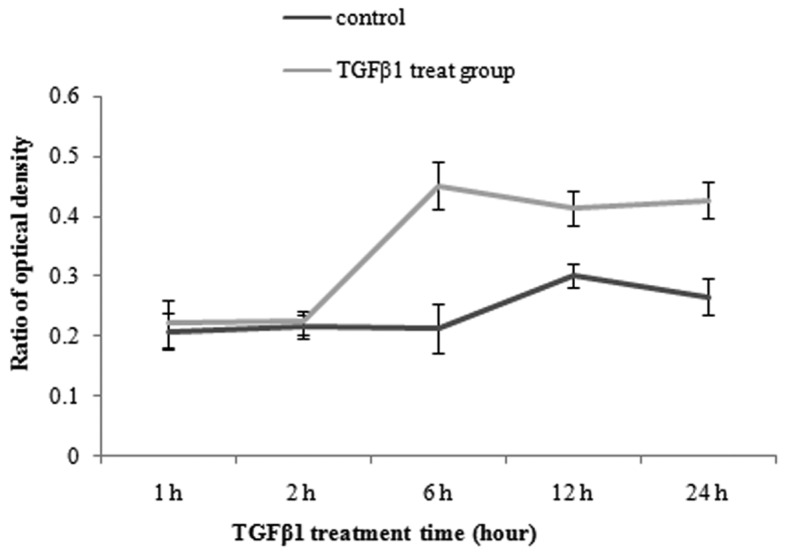
JEG-3 cells were treated with 5 ng/ml TGF-β1, except the blank and control groups. Cellular proliferation was determined using the MTT assay at 1, 2, 6, 12 and 24 h, respectively (mean ± SD, n=3). TGF-β1, transforming growth factor β1.

JEG-3 cells were incubated in six-well plates with an initial concentration of 5×10^4^ cells/ml for 48 h. Wells were divided into six groups as follows: Control, TGF-β1, 1-μM SB203580, 3-μM SB203580, 1-μM LY364947 and 3-μM LY364947 groups. Cells in the control group were cultured with RPMI-1640 only, while the cells in the TGF-β1 groups were treated with 5 ng/ml TGF-β1 and incubate for 2 h. When the cells reached ~80% confluency, they were pretreated with the appropriate concentration of TGF-β1 receptor inhibitor (LY36494; Sigma-Aldrich) and p38 MAPK inhibitor (SB203580; Sigma-Aldrich), and cultured for 2 h. The cells were cultured for 2 h as according to the MTT results (Fig. 1), no obvious effect on proliferation was identified with the treatment of TGF-β1 for 2 h, ensuring that the expression changes of the TGF-β receptors and Smad3 were not affected by changes in cell proliferation levels. Subsequently, 5 ng/ml TGF-β1 (PeproTech, Inc.) was added to each well, with the exception of those in the control group, and incubation was continued for 2 h. Total RNA extraction of JEG-3 cells from each group was performed with TRIzol reagent (Invitrogen Life Technologies, Carlsbad, CA, USA). RNA (1 μg) was reverse transcribed using an M-MLV First-Strand cDNA Synthesis kit (Invitrogen Life Technologies) and random oligodeoxynucleotide primers.

qPCR amplifications were performed using SYBR premix (Invitrogen Life Technologies) for TβRI, TβRII and Smad3. β-actin mRNA was employed as an internal control. The primer sequences used are listed in Table I. The cycling conditions were as follows: 40 cycles of 95°C for 30 sec, 95°C for 5 sec, 95°C for 30 sec and 72°C for 30 sec, followed by 95°C for 1 min, 95°C for 30 sec and 75°C for 30 sec. The obtained results of the mRNA copy number were recalculated per 1 μg of total RNA. Each run was completed using melting curve analysis to confirm the specificity of the amplification and the absence of the primer dimers. All data were quantified by the use of the comparative cycle threshold method, normalized to β-actin.

### Statistical analysis

All data are expressed as the mean ± standard deviation. One-way analysis of variance was used to compare differences among groups. The Student-Newman-Keuls test was performed to assess the differences between pairs of groups. Differences were considered statistically significant at values of P<0.05. All statistical analysis was performed using SPSS 19.0 software (SPSS, Inc., Chicago, IL, USA).

## Results

### Effect of TGF-β on choriocarcinoma cellular proliferation

JEG-3 cells were treated with the same concentration of TGF-β1 at 5 ng/ml, and then cellular proliferation was determined by MTT assay at different time points. As shown in Fig. 1, JEG-3 cells were not significantly affected by the presence of TGF-β at 1 and 2 h, compared with control group. This suggested that TGF-β did not promote the proliferation of choriocarcinoma before 2 h. By the time of 6 h, TGF-β1 exhibited a marked effect on JEG-3 cell proliferation. Additionally, the viability of JEG-3 cell proliferation was gradually decreased by TGF-β1 at 12 and 24 h(Fig. 1).

### Effect of p38 MAPK inhibition on the transcriptional levels of Smad3 in the JEG-3 cell line

**Figure 2:**
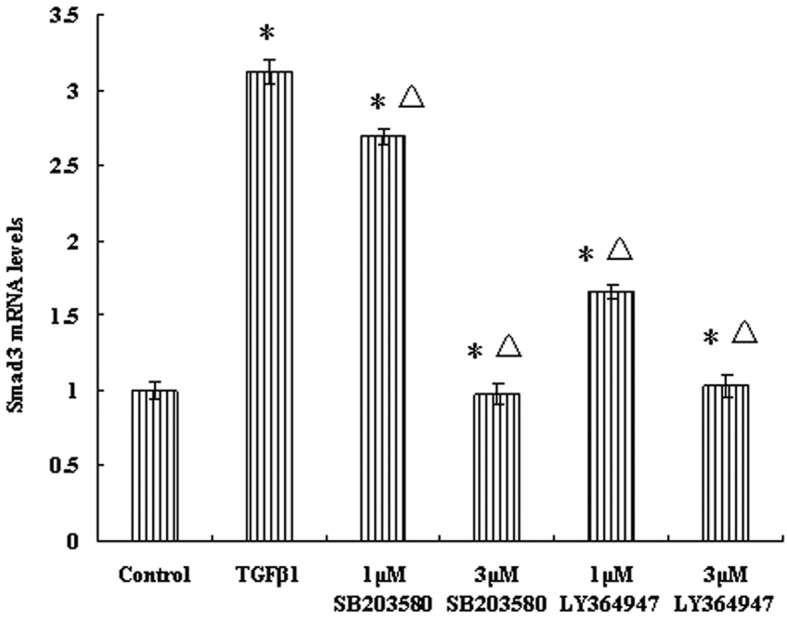
Effect of p38 MAPK and TGF-β1 receptor inhibitors (SB203580 and LY36494, respectively) on the transcriptional levels of Smad3 in the JEG-3 cell line. Cells were divided into six groups: Control group, TGF-β1 group, 1-μM SB203580, 3-μM SB203580, 1-μM LY364947 and 3-μM LY364947 groups. Cells were pretreated with different concentrations of p38 MAPK and TGF-β1 receptor inhibitors, and cultured for 2 h. Subsequently, 5 ng/ml TGF-β1 was added to cells in all groups except the control group, and incubation was continued for 2 h. The mRNA expression levels of Smad3 were determined by reverse transcription quantitative real-time polymerase chain reaction. The data are presented as the mean ± SD (P<0.05). The results are representative of at least three independent experiments. MAPK, mitogen-activated protein kinase; TGF-β1, transforming growth factor β1. ^*^P<0.05 vs. control group; ^Δ^P<0.01 vs. TGF-β1 group.

To examine the roles of Smad3 in the TGF-β pathway and p38 MAPK, the transcriptional levels of Smad3 were examined using the p38 MAPK inhibitor (SB203580) and TGF-β1 receptor inhibitor (LY36494). Following this, 5 ng/ml TGF-β1 was added into each well, except those of the control group, and incubation was continued for 2 h. Previous results from our laboratory indicated that p38 MAPK inhibitors can attenuate TGF-β1-induced Smad3 protein expression (21); thus; we further examined the effect of p38 inhibitors on the transcriptional levels of Smad3 in response to TGF-β1. Compared with the control group, the mRNA expression levels of Smad3 were significantly elevated in TGF-β1 group (P<0.05) (Fig. 2). The results revealed that TGF-β1 promotes Smad3 transcription. With an increasing concentration of inhibitors, the Smad3 transcriptional levels in the LY364947 and SB203580 groups gradually reduced compared with the other two groups (P<0.05) (Fig. 2).

### Effect of p38 MAPK inhibition on TβRI and TβRII transcriptional levels in the JEG-3 cell line

**Figure 3:**
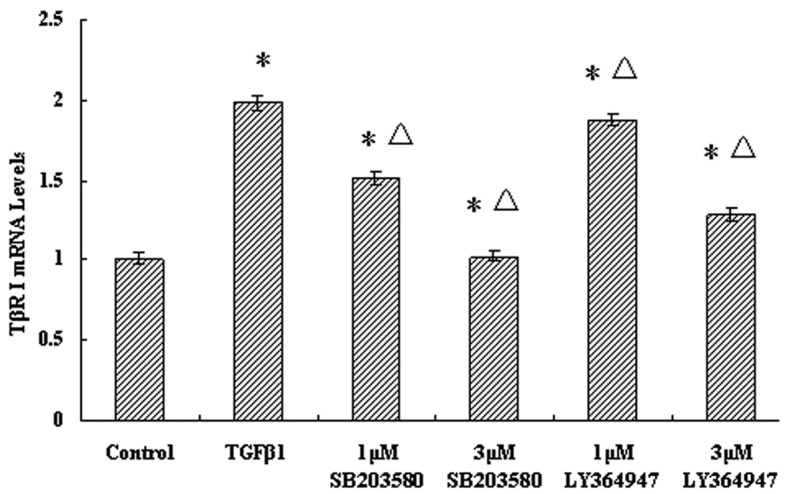
Effect of p38 MAPK inhibition on TβRI transcriptional levels in the JEG-3 cell line. Cells were divided into six groups: Control group, TGF-β1 group, 1-μM SB203580, 3-μM SB203580, 1-μM LY364947 and 3-μM LY364947 groups. Cells were pretreated with different concentrations of p38 MAPK and TGF-β1 receptor inhibitors, and cultured for 2 h. Next, 5 ng/ml TGF-β1 was added to cells in all groups except the control group, and incubation was continued for 2 h. The mRNA expression levels of TβRI were determined by reverse transcription quantitative real-time polymerase chain reaction. Results are presented as the mean ± SD from at least three independent experiments (P<0.05). MAPK, mitogen-activated protein kinase; TGF-β1, transforming growth factor β1; TβRI, TGF-β receptor type I. ^*^P<0.05 vs. control group; ^Δ^P<0.01 vs. TGF-β1 group.

In order to examine the roles of TβRI and TβRII in the regulation of p38 MAPK, the TβRI and TβRII transcriptional levels were examined by blocking p38 MAPK using a p38 MAPK inhibitor, SB203580. The mRNA expression levels of TβRI and TβRII in the TGF-β1 group were both increased compared with those in the control group (P<0.05). Pretreatment with LY36494, a TGF-β-1 inhibitor, resulted in significant decrease (P<0.05) in the mRNA levels of TβRI, in a dose-dependent manner, compared with those of the TGF-β1 group. In the groups treated with SB203580, the trend of TβRI transcriptional levels was similar to that in the LY36494-treated groups, which indicated that the p38 MAPK inhibitor downregulates the TβRI transcriptional level (P<0.05) (Fig. 3).

**Figure 4:**
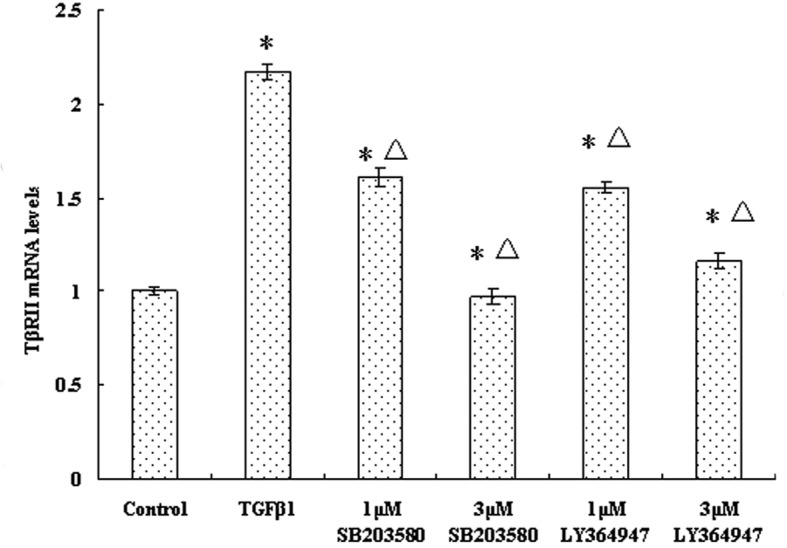
Effect of p38 MAPK inhibition on TβRII transcriptional levels in the JEG-3 cell line. Cells were divided into six groups: Control group, TGF-β1 group, 1-μM SB203580, 3-μM SB203580, 1-μM LY364947 and 3-μM LY364947 groups. Cells were pretreated with different concentrations of p38 MAPK and TGF-β1 receptor inhibitors, and cultured for 2 h,. Following this, 5 ng/ml TGF-β1 was added to cells in all groups except the control group, and incubation was continued for 2 h. The mRNA expression levels of TβRII were determined by reverse transcription quantitative real-time polymerase chain reaction. Results are presented as the mean ± SD from at least three independent experiments (P<0.05). MAPK, mitogen-activated protein kinase; TGF-β1, transforming growth factor β1; TβRI, TGF-β receptor type II. ^*^P<0.05 vs. control group; ^Δ^P<0.01 vs. TGF-β1 group.

The TβRII mRNA expression results were similar to those for TβRI (Fig. 4). The mRNA levels of TβRII were reduced in both the LY36494- and SB203580-treated groups. As the concentration of inhibitor increased, the mRNA levels of TβRII gradually decreased (Fig. 4). The results revealed that blocking the p38 MAPK pathway can modulate the TβRII transcriptional level induced by TGF-β1 in JEG-3 cells.

## Discussion

Choriocarcinoma is a fast-growing and highly malignant tumor. Although the availability of chemotherapy has made the prognosis highly favorable (25), numerous patients cannot tolerate the toxicity and side effects. Therefore, there is an urgent requirement to explore the mechanism of development of choriocarcinoma for new molecular target therapy. TGF-β appears to be a key factor in the development of choriocarcinoma. Any mutations that occur in the components of the TGF-β/Smad signaling pathway can cause the formation of tumors. For example, TβRII has been found to be overexpressed in a bladder cancer cell line, concomitant with point mutations, particularly the Glu269 to Lys mutation (G to A) (26). The mutations of TβRII, which promoted tumor cell growth, did not affect Smad2/3 binding.

In the process of tumor transformation, TGF-β plays two conflicting roles of a tumor suppressor and a tumor promoter. In the early stage of cancer development, TGF-β is anti-proliferative or works as a tumor suppressor, whereas in the late stage it functions as a tumor promoter, involved in metastasis (27–29). In the present study, MTT assay was used to examine the effects of TGF-β1 on JEG-3 cell proliferation. The viability of JEG-3 cell proliferation was tested at 1, 2, 6, 12 and 24 h, and the results demonstrated that TGF-β-1 was able to promote the proliferation of choriocarcinoma. It was also found that, following treatment with TGF-β1, the transcriptional levels of Smad3, TβRI and TβRII were all elevated compared with the control group (Figs. 2–4). This suggested that TGF-β1 can also activate the TGF-β/Smad signaling pathway in choriocarcinoma and the extracellular signal is successfully transmitted into cytoplasm.

TGF-β not only transmit its signal via Smad proteins, but can also activate other signaling molecules such as p38 MAPK (30). A study by Daroqui *et al* demonstrated that p38 MAPK and MEK contribute to TGF-β stimulation of cell motility and invasion by analyzing signal transduction mediators. Additionally, both the MAPK-dependent and -independent pathways are necessary for TGF-β-induced effects (31). According to a study by Gui *et al*, the prolonged and sustained activation of the p38 MAPK pathway requires Smad signaling, which is observed in hepatocytes, osteoblasts and pancreatic carcinoma cells. Smad activation induces the expression of GADD45β, an upstream activator of MKK4, and thus promotes the prolonged activation of p38 MAPK (32).

The TGF-β receptors are gateways to the TGF-β/Smad pathway, which aids the extracellular signal to cross the membrane into the cytoplasm (33). According to a study by Huang and Chen, TβRII alone is able to mediate TGF-β signaling to ERK1/2, and differences in the level of TβRII expression determine whether or not TGF-β activates or inhibits ERK1/2 (6). It has been suggested that inhibiting the activity of TGF-β receptor blocks TGF-β-induced MAPK activation. Dalliher *et al* demonstrated that TGFβII mutants in breast cancer cells completely abrogated p38 MAPK activation induced by TGF-β, but failed to affect TGF-β stimulation of Smad2/3 (34). A study by Ohshima showed that mutant TGFβI did not affect activation of the Smad pathway, but retained signaling via the MAP kinase pathway (35). The study also suggested that TGF-β receptor-activated p38 is involved in TGF-β-induced apoptosis but not growth arrest in mouse mammary gland epithelial cells. Based on these observations, the current study attempted to investigate the effect of crosstalk between TGF-β/Smad and p38 MAPK signaling on the expression of TGF-β receptors and Smad3 in choriocarcinoma (JEG-3) cells. Cells were pretreated with different concentrations of TGF-β1 receptor inhibitor and p38 MAPK inhibitor, respectively, and incubated for 2 h. Subsequently, 5 ng/ml TGF-β1 was added to the cells and cultured for 2 h. According to the MTT assay results, no obvious effect on proliferation was observed with treatment of TGF-β1 for 2 h, so 2 h was selected as the duration of TGF-β1 treatment to ensure that the expression changes in TGF-β receptors and Smad3 were not affected by changes in cell proliferation levels. The expression of TβRI, TβRII and Smad3 was reduced in a dose-dependent manner in the 1- and 3-μM LY364947 groups, compared with the control group. This indicated that the TGF-β receptor inhibitor was able to inhibit the TGF-β/Smad signaling pathway to an extent. Additionally, in the 1- and 3 μM SB203580 groups, the trends of TβRI, TβRII and Smad3 transcriptional levels were consistent with those in the LY364947 treatment groups. p38 MAPK inhibitors can attenuate TGF-β1-induced TβRI, TβRII and Smad3 transcriptional levels. These results revealed that the TGF-β/Smad signaling pathway may be affected by the p38 MAPK pathway, and blockade of the p38 MAPK pathway can downregulate the activated TβRI, TβRII and Smad3. This suggests that diverse biological responses regulated by TGF-β are mediated not only via Smad proteins, but also by different downstream R-Smad-independent signaling pathways. Any changes that occur in these downstream signaling pathways may have an effect on the genesis or progression of choriocarcinoma. Further clarification of the mechanisms of the crosstalk between the TGF-β and p38 MAPK pathways in cell models may offer novel breakthroughs and potential applications in the field of therapeutic approaches.

## Figures and Tables

**Table I:** PCR primers used in reaction.

Target	Primer	Sequence (5′-3′)	Length of amplicon (bp)	Tm (°C)
TβRI	Forward	GCAGTAAGACATGATTCAGCCACAG	190	58.1
	Reverse	CAATGGAACATCGTCGAGCAA		
TβRII	Forward	GAAATTCCCAGCTTCTGGCTCA	143	57.2
	Reverse	CTGTCCAGATGCTCCAGCTCAC		
Smad3				
	Forward	CCAGGGCTTTGAGGCTGTCTA	143	59.2
	Reverse	GCAAAGGCCCATTCAGGTG		
β-actin	Forward	TGGCACCCAGCACAATGAA	186	56.0
	Reverse	CTAAGTCATAGTCCGCCTAGAAGCA		
